# Traditional Wisdom for Modern Sustainability: A Dish-Level Analysis of Japanese Home Cooking in NHK Today’s Cooking

**DOI:** 10.3390/nu17162712

**Published:** 2025-08-21

**Authors:** Rui Fu, Yasuhiro Yamanaka

**Affiliations:** 1 Graduate School of Environmental Science, Hokkaido University, Hokkaido 060-0810, Japan; 2 Faculty of Environmental Earth Science, Hokkaido University, Hokkaido 060-0810, Japan; galapen@ees.hokudai.ac.jp

**Keywords:** Sustainable Healthy Diets (SHDs), Japanese cuisine, dietary transitions, dish-level analysis, nutritional diversity, environmental sustainability, cultural continuity

## Abstract

Background: Balancing nutrition security with environmental sustainability is a key priority in global food policy, with Sustainable Healthy Diets (SHDs) serving as a critical framework aligned with the UN Sustainable Development Goals (SDGs). Traditional Japanese cuisine reflects SHD principles through its emphasis on plant-based, seasonal, and minimally processed dishes. However, modern, globalized dietary patterns increasingly feature ultra-processed foods, raising concerns about health risks such as high sodium intake. Methods: This study adopts a novel dish-level content analysis of 120 contemporary recipes from NHK Today’s Cooking between 2023 and 2025, a TV program by Japan’s national public broadcaster that is widely regarded as reflecting the practices of Japanese home cooking, to examine how SHDs pillars—nutritional diversity (e.g., varied protein sources), environmental sustainability (e.g., low-carbon ingredients), and cultural continuity (e.g., traditional techniques)—are embedded in Japanese home cooking. Unlike macro-level consumption or nutrition data, this dish-level approach reveals how individual dishes embody sustainability through ingredient selection, preparation methods, and cultural logic. Results: Quantitatively, pork (33.3%) and seafood (19.2%) together dominated main protein sources, with minimal beef (2.5%) and a notable presence of soy-based foods (12.5%), supporting lower reliance on environmentally intensive red meat; mean salt content per person in main dishes was 2.16 ± 1.09 g (28.9% for men, 33.3% for women of Japan’s daily salt targets), while recipe patterns emphasizing fermentation and seasonal alignment highlight possible pathways through which Japanese dietary practices can be considered ecologically efficient. Simultaneously, the analysis identifies emerging challenges, encompassing environmental issues such as overfishing and public health concerns like excessive sodium consumption. Conclusions: By centering dishes as culturally meaningful units, and using media recipes as reproducible, representative datasets for monitoring dietary change, this approach offers a reproducible framework for assessing dietary sustainability in evolving global food systems.

## 1. Introduction

The global food system faces a dual imperative: to ensure adequate nutrition for a growing population while minimizing environmental degradation [1]. These intertwined challenges have elevated the concept of Sustainable Healthy Diets (SHDs) as a central policy framework at the nexus of public health and ecological sustainability, particularly within the scope of the United Nations Sustainable Development Goals (SDGs). Defined by the Food and Agriculture Organization (FAO) and the World Health Organization (WHO), SHDs are “dietary patterns that promote all dimensions of individuals’ health and well-being; have low environmental pressure and impact; are accessible, affordable, safe and equitable; and are culturally acceptable” [2]. This integrative model seeks to reconcile nutritional adequacy, environmental stewardship, and cultural viability.

Traditional Japanese dietary practices, particularly those classified under washoku, have gained international recognition for their alignment with the principles of SHDs. Emphasizing moderation, variety, seasonality, and balance, washoku prioritizes plant-based ingredients, minimal processing, and the use of local and seasonal foods—characteristics closely associated with both favorable health outcomes and a reduced environmental footprint [3]. In recognition of its holistic approach to food, washoku was inscribed on the United Nations Educational, Scientific and Cultural Organization (UNESCO) Intangible Cultural Heritage list in 2013, as a comprehensive system of knowledge, practice, and ethics grounded in respect for nature and sustainable resource use [4]. What sets this inscription apart is its exceptional national scope. Washoku’s UNESCO inscription is notable for its rare national scope, placing it alongside the “gastronomic meal of the French” and “traditional Mexican cuisine” as one of the few food-related recognitions that acknowledge an entire national dietary culture [5].

As defined by the Ministry of Health, Labour and Welfare (MHLW), the dietary format most closely associated with washoku is ichiju-sansai, consisting of one soup, one main dish, two side dishes, and a staple grain, typically rice [6]. This configuration exemplifies a nutritionally balanced and resource-efficient approach that aligns with both modern public health goals and ecological priorities. However, contemporary Japanese dietary practices have been reshaped by the forces of globalization, urbanization, and shifting consumer preferences. The expansion of convenience stores, fast-food chains, and ready-made meals has introduced a growing array of ultra-processed and Western-style foods into the daily diet [7].

While washoku continues to serve as a cultural ideal and reference point, it is not without nutritional limitations. A prominent concern is the traditionally high sodium content of many Japanese dishes. Excessive sodium intake has been consistently linked to elevated risks of hypertension and cardiovascular disease [8]. In 2010, Japan’s average daily salt intake was 12.7 g—substantially higher than that of the United States (9.2 g/day)—and that of Western Europe averaged 9.7 g/day [9]. Although recent decades have seen gradual improvements, national surveys still indicate that average intakes exceed recommended limits [10]. In response, public health initiatives such as “Health Japan 21” have been implemented to promote salt reduction through educational campaigns, product reformulation, and improved nutrition labeling [11].

Simultaneously, the actual dietary landscape in Japan has become increasingly diverse and internationalized. In addition to traditional staples such as udon, soba, and sushi, many Japanese households now incorporate a wide variety of global dishes into their daily meals. Examples include Chinese-style mapo tofu, Korean *chijimi* (savory pancakes), Japanese-style adaptations of ramen, and Western imports like pizza and hamburgers. While such culinary diversification can enhance dietary variety and consumer enjoyment, it also introduces new public health and environmental challenges. The increasing reliance on ultra-processed foods and the declining prevalence of home-cooked meals have been associated with rising rates of obesity, hypertension, and other non-communicable diseases, as well as greater environmental impacts linked to resource-intensive food production [12,13]. These developments call for a renewed examination of the relevance of traditional Japanese dietary practices in the context of contemporary SHDs frameworks.

Macro-level consumption data offer important insights into the broader nutritional transitions shaping the Japanese food system. For example, while per capita beef intake in Japan remains relatively modest compared to other countries in the Organisation for Economic Co-operation and Development (OECD), total meat consumption has steadily increased over recent decades and now approximates European averages. At the same time, Japan maintains a relatively high level of seafood consumption, reflecting both historical continuity and contemporary dietary preferences [14,15]. These aggregated figures—comprising millions of individual food choices—provide valuable indicators of dietary transition at the national level.

Nevertheless, these national averages have limitations in illuminating how dietary changes unfold in real daily life. They may not fully capture how meals are selected and experienced in everyday domestic settings, nor do they reflect the cultural meanings, preparation methods, or social dynamics embedded in daily cooking practices. Understanding the actual composition of meals—as experienced in household kitchens and reflected in widely consumed recipes—is essential to grasp the nuanced transformations of Japanese dietary practices. Investigating the lived realities of what people cook and eat thus provides a more grounded and culturally informed perspective, complementing broad statistical trends with specificity.

Previous research on Japanese dietary practices, including washoku, spans multiple disciplinary domains. Nutritional studies emphasize the health benefits of traditional Japanese diets, citing reduced risks of chronic diseases linked to high consumption of vegetables, fish, and fermented foods [1,3]. Ethnographic and historical studies highlight umami and *dashi* as central to Japanese culinary tradition, showing how cultural and scientific factors have shaped changing perceptions of glutamate, from a key sensory element to a scientifically recognized flavor enhancer [16]. Scholars have also investigated the globalization of Japanese food—especially sushi—highlighting how the Japanese state and media leverage notions of authenticity and culinary nationalism to reassert cultural ownership amid the proliferation of hybridized international forms [5,17]. Policy-oriented studies focus on national initiatives such as “Healthy Japan 21” and the “Japanese Food Guide Spinning Top,” which aim to address rising rates of non-communicable diseases and declining food literacy through structured dietary education [18,19,20]. In the field of economics, research demonstrates that the global diffusion of Japanese food culture—proxied by indicators such as online searches for sushi—positively influences exports of related products like sake, revealing a cultural spillover effect in international trade [21]. These studies construct a multifaceted understanding of Japanese foodways as a dynamic interplay between health, culture, policy, and globalization.

However, the previous research lacks robust methodologies to systematically evaluate how the three interconnected pillars of sustainable diets—nutritional diversity, environmental sustainability and cultural heritage preservation—are operationalized, balanced, or compromised in routine household food practices.

To address this question, we conduct a dish-level content analysis of Today’s Cooking, a long-running culinary television program produced by NHK, Japan’s national public broadcasting organization. This program holds unparalleled sociological significance as a de facto archive of mainstream Japanese domestic food practices since 1957. Officially recognized by Guinness World Records as the world’s longest-running television cooking program (67 years as of November 2024), Today’s Cooking has aired approximately 15,500 episodes and introduced over 46,600 distinct recipes [22,23]. Its development closely reflects Japan’s broader socio-demographic and nutritional transitions—beginning with efforts to combat postwar malnutrition, adapting to rising economic affluence, evolving household structures (evidenced by portion size reductions from five to four to two servings), and responding to shifting public health concerns, such as decreasing the salt content in pickled plums from 20% to 5% [22,23]. In celebrating its 60th anniversary, NHK Today’s Cooking reflected on its mission as a public broadcaster, emphasizing the importance of providing recipes that go beyond catering to fleeting popular trends. According to the program’s creators, the intention is to highlight seasonality, nutritional balance, and the cultural and natural context of food using seasonal ingredients. In addition, recognizing the decline in traditional television viewership and the rise of internet-based information sources, the program has sought to maintain relevance by updating its website with recent recipes [24,25]. This approach preserves the educational and cultural values embedded in Japanese culinary traditions and illustrates how these values are represented in contemporary media. Previous studies and conference reports, using the monthly NHK paper media, have documented long-term trends, including a gradual decline in salt content [26,27], suggesting that the program may serve as a useful archive for examining dish-level dietary patterns over time. According to the Japan Magazine Publishers Association, between October 2023 and September 2024, the combined monthly circulation of Today’s Cooking and Today’s Cooking Beginners was 299,117 copies, the highest among Japanese monthly cooking magazines (second place: Lettuce Club, 183,159 copies) [28]. In addition, NHK’s Instagram account for the program has approximately 167,000 followers [29], comparable to Japan’s representative recipe-sharing site Cookpad with 193,000 followers [30].

As a highly trusted NHK production, the program offers a uniquely comprehensive dataset for examining how principles of sustainable and healthy diets are embedded—or compromised—in daily practice. By reviewing this extensive recipe corpus, all 120 recipes featured on the program’s homepage between April 2023 and April 2025, we evaluate key indicators of sustainability, including nutritional diversity (e.g., ingredient variety, protein source composition), environmental impact (e.g., frequency of high-carbon ingredients), and cultural continuity (e.g., persistence of traditional seasonings and preparation methods). This empirical approach enables us to trace how the three pillars of sustainable diets—health, environmental sustainability, and cultural relevance—have been negotiated and transformed over time within the lived realities of Japanese home cooking.

The methodological innovation of this study lies in reframing dietary assessment around prepared dishes rather than abstract food groups or nutrient counts. Dishes may serve as culturally legible, nutritionally dense, and environmentally meaningful units with the potential to provide insights into household dietary practices. This dish-level focus offers a framework for exploring how macro-level dietary trends might be reflected in home cooking practices. The framework also holds cross-cultural relevance, offering a model for examining dietary transitions across national contexts.

This study makes three key contributions. First, it provides an empirically grounded portrait of contemporary Japanese eating patterns by analyzing recipes featured on NHK Today’s Cooking, a nationally representative media corpus. This may reveal concrete patterns in ingredient use and meal composition relevant to real-world practices. Second, it illustrates how traditional dietary forms coexist with globalized culinary influences, challenging static notions of “authentic” Japanese food. By tracking recipe genres and incorporating foreign-origin dishes, we show how Japanese food culture negotiates hybridization while retaining distinct cultural markers. Third, it advances a practice-oriented approach to dietary sustainability, demonstrating how daily cooking mediates cultural continuity, nutritional adequacy, and environmental concerns. Assessing recipes by sustainability indicators—such as protein diversity, salt levels, and seasonality—links household practices to broader SHDs frameworks.

## 2. Materials and Methods

This study draws on a dataset of 120 recipes featured on the official website of NHK Today’s Cooking between April 2023 and April 2025. The NHK Today’s Cooking creators indicate on the program’s official website that the platform serves as an entry point for introducing its current recipes [24,25]. The website operates as a rolling two-year archive, with older recipes replaced by new ones and no longer publicly accessible. Accordingly, all 120 recipes from this archive were collected for analysis, representing the current repertoire of Today’s Cooking, which may provide insights into patterns emphasized in contemporary Japanese home cooking. The sample includes a balanced representation of main dishes, side dishes, soups, and desserts, thus capturing the structural diversity of everyday Japanese meals. As an example, a screenshot of one complete recipe page, including all available information, is provided in the Supplemental Material. From all 120 dishes, the data were coded to construct a dataset containing dish name, episode date, ingredients, cooking time, cooking steps, salt content(g) and calorie content (kcal) per serving.

For each recipe, structured coding was applied across four main dimensions:

- Protein Source: Ingredients were classified based on their primary protein content (e.g., plant-based, seafood, poultry, red meat). When multiple protein sources were present, the dominant source was determined by the proportion of each protein source by weight (grams). Dishes with multiple significant protein inputs were categorized as composite-protein recipes. The representativeness of these categories was then verified by comparison with data from the Ministry of Agriculture, Forestry and Fisheries (MAFF).
- Traditional and Cultural Seasonings: Seasonings were grouped into fermented (e.g., miso, soy sauce, mirin, sake, rice vinegar) and non-fermented types (e.g., salt, sugar, pepper) based on established culinary references [16,31], with coding conducted by the first author and cross-checked by the second author, with discrepancies discussed and resolved to ensure consistency. Frequency counts for each seasoning category were recorded to evaluate reliance on traditional flavor profiles. Consistency with traditional washoku principles was then assessed.
- Cooking Methods: Instructional text from each recipe was analyzed for high-frequency cooking verbs (e.g., boiling, stir-frying, pan-frying, steaming, deep-frying, mixing), which served as indicators of dominant cooking techniques.
- Nutritional and Cultural Indicators: Where reported, salt content was recorded as a proxy for nutritional health. Additional variables—such as seasonal ingredient use and estimated cooking time—were included to assess cultural embeddedness and everyday practicality. It should be noted that only recipes with clearly defined serving sizes were included, while those labeled with ambiguous portion descriptions such as “easy-to-make quantity” were excluded due to the inability to perform accurate calculations. For eligible recipes, the salt content was taken directly from the nutritional information provided on the official NHK recipe website, where values are standardized and reported on a per-person basis. Additionally, non-dish items such as desserts were excluded from the dataset to maintain analytical focus on main dishes, which better represent typical daily meals and their associated salt content in Japan. The obtained salt values were compared with WHO and MHLW guidelines to verify alignment with recommended intake levels.

Full recipe data and the corresponding coding details are provided in the Supplementary Material.

## 3. Results

### 3.1. Protein Sources and Meat Consumption Patterns

The 120 dishes featured in NHK Today’s Cooking were classified into six primary categories based on their main ingredients: pork (*n* = 40), chicken (*n* = 21), beef (*n* = 3), fish and seafood (*n* = 23), meatless (*n* = 19), and non-dishes (*n* = 18), the last comprising items such as desserts and seasonings (Table 1). This reflects core features of the Japanese diet, including the predominance of pork and fish and the notably limited representation of beef.

**Figure 1:**
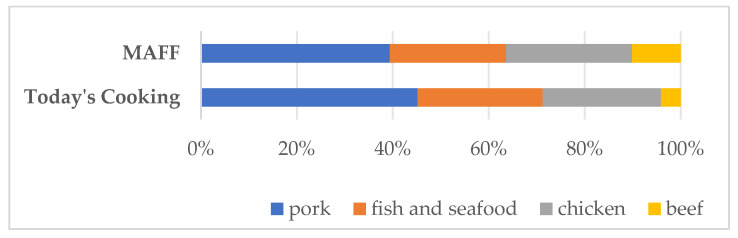
Distribution of protein sources in NHK Today’s Cooking recipes (percentage of total dishes) and in national average daily per capita intake (grams per day), based on the 2022 Food Balance Sheet [32]. Note: Meatless and non-dish categories are excluded from this figure.

**Figure 2:**
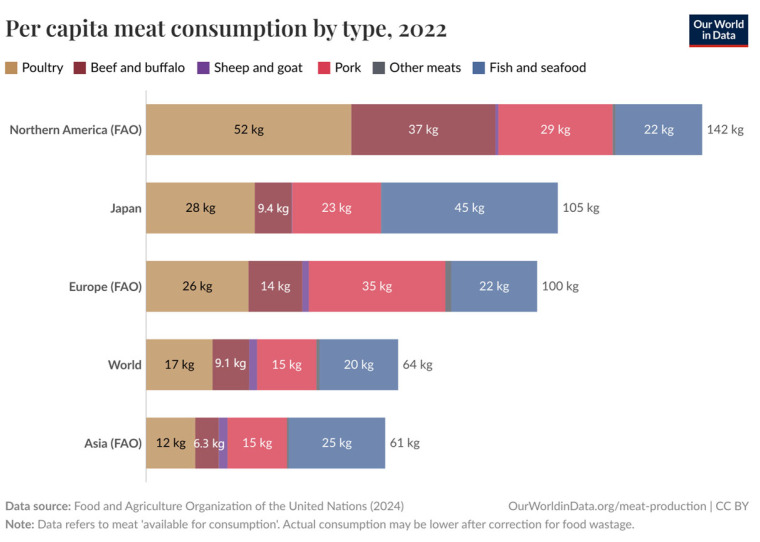
Per capita meat and seafood consumption by type (kg/year): Comparison of Japan with North America, Europe, Asia, and Global Averages (FAO Data) [14].

A comparison between the dish-level protein source proportions in NHK Today’s Cooking recipes and population-level data from the survey conducted by MAFF in 2023 reveals a close alignment in the relative distribution of protein sources (Figure 1). Although it is difficult with direct quantitative comparison due to variations in portion sizes and meat content per dish, this close alignment means the representativeness of our dataset. The same meat consumption trends—low beef intake and high seafood consumption relative to western countries—observed both in the MAFF population-level data and the NHK recipe dataset are confirmed in cross-regional data from FAO (Figure 2).

### 3.2. Plant-Based Protein Sources

Beyond animal proteins, the Japanese diet extensively incorporates legumes—particularly soy-derived products such as tofu, natto, and miso—as nutritionally significant sources of plant-based protein deeply embedded in daily food culture. We found a total of 15 unique preparations, categorized by legume type and protein pairing (Table 2). Soy products dominate the legume category, with tofu featured in five distinct dishes: combined with beef (*n* = 1), chicken (*n* = 1), seafood (*n* = 1), and a fully meatless dish (*n* = 2). Natto, a fermented soybean product, appears in one dish paired with pork. Miso, another fermented soy derivative, is used in seven distinct dishes: two with pork, two with seafood (one of which also contains tofu), two meatless dishes (including one that also incorporates tofu), and one non-dish recipe used to prepare seasoning. To avoid double counting, two overlapping dishes containing both tofu and miso are counted under tofu only. Beyond soy, other legumes also contribute to dietary diversity. We identified four supplementary non-soy legume-based dishes: two with green beans, one with broad beans, and one with white kidney beans. This comprehensive incorporation of leguminous ingredients, particularly soy products, underscores the functional role of plant-derived proteins in traditional Japanese dietary patterns.

### 3.3. Fermented and Non-Fermented Seasonings

In addition to their role as plant-based protein sources, legumes—particularly soybeans—also serve as the foundation for a range of traditional fermented seasonings that are indispensable to Japanese culinary practice. The seasoning usage reveals distinct patterns between fermented and non-fermented ingredients in Japanese cooking (Table 3). Non-fermented seasonings such as salt (*n* = 72), pepper (*n* = 56), and sugar (*n* = 39) were the most frequently used, reflecting their foundational role in basic taste adjustment. However, fermented seasonings also appeared prominently, led by soy sauce (*n* = 52), which contributes depth of flavor through its characteristic umami and savory profile.

Sake or cooking wine (*n* = 47) was the most frequently used seasoning, contributing depth and umami across a wide range of preparations. Vinegar (*n* = 23) commonly added acidity and balance, particularly in cold dishes and pickles. Mirin (*n* = 22), a sweet rice wine, imparted mild sweetness and richness, especially in simmered dishes. Miso appeared less frequently (*n* = 7) but served a distinct role as a concentrated seasoning base, particularly in soups. Together, these findings highlight the significant role of fermented seasonings in shaping the overall flavor complexity of Japanese cuisine. While used less often than salt or pepper, their inclusion reflects a deep culinary tradition that values layered, balanced flavors, often achieved through fermentation-derived depth and subtlety.

### 3.4. Pickled Foods in Traditional Practices

Although pickled items (*tsukemono*) were present in only four recipes, their inclusion highlights a traditional strategy for food preservation that contributes meaningfully to sustainability in Japanese culinary practices. As shown in Table 4, these pickled preparations employ natural preservation agents such as salt, alcohol, vinegar, and sugar, extending shelf life significantly while maintaining palatability and nutritional value. Notably, honey-pickled and traditionally salted plums (*ume*) can be stored for up to one year, demonstrating a preservation approach that reduces food waste and supports year-round ingredient availability. Even items with shorter shelf lives, such as the Taiwanese tea-infused plum, offer culturally distinctive flavors while illustrating diverse methods of fermentation and preservation.

### 3.5. Health-Driven and Environmentally Sustainable Cooking Methods

In line with the sustainable principles reflected in traditional preservation techniques, the cooking methods featured in the dataset also suggest a preference for environmentally conscious practices (Table 5).

Among the 102 recipes, 174 instances of cooking methods were recorded, as individual recipes often employed multiple techniques. The most prevalent methods were simmering or boiling (54 mentions) and pan-frying (42), followed by stir-frying (37) and steaming (18). Less frequently used were deep-frying (10), no-cooking or reheated (9), baking (2), and pickling (2). The techniques of simmering/boiling, steaming, no-cooking/reheating, or pickling were intentionally employed, with 74 of 102 dishes using at least one of these methods (a simple binary choice of whether or not these techniques were used, with a χ^2^ test yielding *p* = 0.01).

This distribution reflects a culinary emphasis on methods generally regarded as healthier and more sustainable. Techniques such as simmering, steaming, and no-cooking approaches typically require less added fat and energy-intensive processing, aligning with dietary practices that promote cardiovascular health and reduce environmental impact [33]. The low frequency of deep-frying and heavy processing further suggests an alignment with public health goals. These findings support the interpretation of NHK Today’s Cooking as a media platform that not only preserves culinary tradition but also subtly reinforces health-conscious and ecologically considerate food preparation methods.

### 3.6. Seasonal Alignment in Ingredient Selection and Culinary Practice

Each recipe explicitly specifies its associated season, reflecting a deliberate emphasis on seasonality (*shun*), a core principle in traditional Japanese cuisine. This seasonal orientation is evident not only in the titles of the dishes but also in their ingredient compositions. For example, dishes such as “Spring Cabbage and Pork with Clam Steamed” (29 March 2023), “Pork and Spring Cabbage Oil Steamed” (2 April 2025), “Steamed New Onion” (26 May 2023), and “Korean-Style New Potato” (7 April 2023) prominently feature early-season vegetables like spring cabbage, new onions, and new potatoes. Similarly, “Summer Vegetable Mixed Pickles” (12 July 2023) include a diverse assortment of summer produce—cucumber, carrot, celery stalks, red and yellow bell peppers, okra, Japanese ginger (*myoga*), and ginger—corresponding with their peak seasonal availability. “Autumn Eggplant and Minced Meat Simmered Dish” (15 September 2023) exemplifies the use of autumn-specific ingredients, further illustrating a consistent pattern of seasonal alignment. Temporal ingredient utilization identifies cucumber as exhibiting peak occurrence in recipes from June through October, aligning with its classification as a quintessential summer vegetable in Japanese food culture. Lotus root demonstrates peak utilization from September to November, corresponding to its established cultural association as a representative autumn ingredient in Japan. These patterns highlight the intentional integration of seasonally characteristic vegetables within culinary practices. Taken together, these findings validate an ingredient selection framework that is intricately aligned with natural seasonal cycles. This not only underscores a cultural appreciation for seasonal freshness but also demonstrates an adherence to traditional Japanese culinary principles.

### 3.7. Salt Content and Public Health Considerations

Having established the seasonal orientation of ingredient selection, it is also important to evaluate the nutritional implications of these dishes—particularly in terms of salt content, which remains a critical public health concern in Japan. The mean salt content per person across 85 analyzed main dish recipes was 2.16 ± 1.09 g. This represents 28.9% or 33.3% of Japan’s daily salt intake targets set by the MHLW in 2020 (<7.5 g/day for men; <6.5 g/day for women) [34] and 43.3% of the WHO’s stricter guideline (<5.0 g/day) [35]. One recipe exceeded the WHO recommended daily salt intake limit of 5 g: Buta no Shôgayaki Nose Hiyashi Chûka (Chilled Chinese Noodles with Ginger Pork) at 6.2 g (dataset numbers 63; see Supplementary Material). Although each dish accounts for only one of three daily meals, 55 dishes exceeded one-third of the WHO daily salt guideline (1.67 g), while 25 and 35 dishes exceeded one-third of the MHLW daily salt targets for men (2.50 g) and women (2.17 g), respectively. Even when accounting for typical accompaniments (e.g., soups, rice, side dishes), the recipes analyzed here exhibit salt content levels that are substantially below Japan’s population average of 12.7 g/day [9]. Main dishes introduced in Today’s Cooking may anchor salt-reduced diets meeting global health targets.

Reflecting increasing public health awareness of high salt intake issue, NHK Today’s Cooking has featured recipes that promote salt reduction while preserving cultural authenticity. For instance, the program introduced a low-salt version of pickled plum (*umeboshi*) containing only 5% salt—a significant reduction from conventional levels—described as “a user-friendly alternative that balances ease of preparation with reduced salt content, without being overly sweet like commercial honey plums.” Such adaptations show the program’s responsiveness to dietary challenges and its role in promoting healthier versions of traditional Japanese cuisine

This finding reflects a form of intentional culinary adaptation, in which traditional food practices are preserved while aligning with contemporary public health objectives aimed at reducing salt-related health risks in Japan.

## 4. Discussion

By reviewing recipes from NHK Today’s Cooking, this study identifies four interrelated dimensions that reflect the evolving integration of cultural heritage, health promotion, economic efficiency, and environmental responsibility within Japan’s contemporary food practices.

Unlike prior research based on official nutritional surveys or food group statistics issued by agencies such as MAFF, our dish-level analysis captures the nuanced ways in which protein sources, seasonality, and traditional techniques are negotiated in actual meal composition. This approach enables the identification of sustainability indicators—such as low-carbon protein choices and use of fermentation—on a practice-oriented level. It also uncovers dynamics that are invisible in compositional data alone, such as the hybridization of global and local cuisines within a Japanese flavor framework.

### 4.1. Bridging Culinary Heritage and Sustainable Protein Strategies

The protein composition evident in the dataset from Today’s Cooking—dominated by pork (33.3%) and seafood (19.2%), with minimal inclusion of beef (2.5%)—reflects Japan’s distinct dietary ecology shaped by both historical and environmental determinants [36]. Pork and chicken frequently appear in traditional Japanese preparations, while also incorporating Vietnamese and Korean influences, demonstrating regional culinary adaptability. Seafood remains closely aligned with authentic washoku techniques, maintaining traditional preparation methods. In contrast, beef appears in only three dishes, with two adopting foreign formats (Chinese stir-fry and Russian stroganoff) and just one employing domestic method, underscoring its limited cultural integration. Historically, limited arable land and resource constraints favored the production and consumption of less resource-intensive livestock such as pork and poultry, rather than cattle [37]. In recent decades, particular attention has been paid to greenhouse gas (GHG) emissions and land-use efficiency. Accordingly, Japan’s per capita beef consumption remains relatively low at 7 kg/year—approximately 49% of the OECD average—indicating a long-term dietary structure with comparatively low beef intake compared to many other industrialized countries [15].

In 2022, Japan’s total per capita meat consumption was approximately 105 kg annually, of which fish accounted for nearly 45 kg, markedly higher than the global averages of 64 kg total meat and 20 kg fish per person [14]. This elevated reliance on seafood supports health outcomes aligned with SDG 3 (Good Health and Well-being), given fish’s rich nutrient profile including high-quality protein and omega-3 fatty acids [37]. However, the substantial dependence on marine resources presents critical challenges for SDG 14 (Life Below Water). Prolonged fishing pressure and significant seafood imports have contributed to the severe depletion of key fish stocks, undermining marine biodiversity and ecosystem resilience [38,39,40]. This situation creates a sustainability paradox: nutritional benefits are achieved at the expense of marine ecosystem health.

To address this, implementing science-based fisheries management is essential, alongside accelerating the expansion of sustainable aquaculture to meet domestic demand without depleting wild resources. At the same time, promoting alternative proteins, such as plant-based meat substitutes, can reduce pressure on marine ecosystems and diversify diets. These strategies can be supported by Japan’s historically low beef consumption and rich tradition of plant-based foods.

Within this dietary landscape, the presence of traditional soy-based foods—such as tofu, natto, and miso—in approximately 13% of recipes highlights the enduring cultural and nutritional role of plant-derived proteins. These foods are associated with enhanced ecological efficiency and improved nutritional outcomes [41]. Importantly, four soy-inclusive dishes in the dataset also contain animal protein, illustrating a hybridized approach to protein sourcing that balances cultural preference with sustainability considerations.

From an environmental standpoint, the inclusion of soy is particularly consequential. Soy protein production entails significantly lower GHG emissions compared to ruminant-derived protein sources; for instance, life cycle assessments indicate that soy protein emits up to 150 times less CO_2_-equivalents per kilogram of protein than beef [42]. Nutritionally, fermented soy products, a staple of traditional Japanese cuisine, provide all essential amino acids. Microbial fermentation breaks down proteins, enhancing their digestibility and absorption to improve nutritional value [43]. This process reduces the risk of nutrient deficiencies when animal protein intake is limited.

This synergistic pattern—characterized by moderate consumption of non-ruminant animal proteins and the strategic integration of nutrient-dense plant-based sources—offers a referential model for reconciling nutritional adequacy with reduced environmental impact. As such, it provides valuable insights for the development of sustainable dietary frameworks that are both culturally grounded and ecologically responsible.

### 4.2. Culinary Techniques as Health-Sustainability Intermediaries

In the Japan Public Health Center (JPHC) baseline survey encompassing over 140,000 participants, boiling (comparable to simmering) was the predominant cooking method for meat across most regions (excluding Okinawa), followed by grilling and stir-frying; for vegetable dishes, boiling, raw preparation, and stir-frying were most frequently employed [44]. This established pattern closely aligns with the cooking methods observed in our dataset (Table 5). The congruence between these datasets suggests that the culinary techniques captured in our analysis may reflect patterns commonly observed in Japanese cooking practices.

From both sustainability and health perspectives, the dominance of moisture-based methods—particularly simmering (54 mentions) and steaming (18)—is notable. These techniques require less oil and energy, supporting lower greenhouse gas emissions and better nutrient retention than high-heat, oil-intensive methods like deep-frying (10) or baking (2) [45]. Steaming, although not documented in national dietary surveys, appears frequently in the dataset and is associated with minimal nutrient loss, particularly of chlorophyll, soluble proteins, vitamin C, and glucosinolates [46]. Simmering is widely regarded as a gentle cooking method that helps preserve nutrients while requiring relatively low energy input. Stir-frying, when performed quickly with minimal oil, balances flavor enhancement with nutrient retention. The relatively limited use of frying and baking is notable in the dataset and may suggest dietary preferences that are mindful of health or ecological impact.

These findings underscore a culinary logic grounded in moderation and nutrient conservation—principles embedded in traditional Japanese cuisine. The convergence of historical and contemporary data points to a resilient dietary model that not only meets cultural and sensory expectations but also aligns with contemporary public health goals and environmental imperatives.

### 4.3. Seasonality in Ingredients and Sustainable Diets

The explicit seasonal tagging of recipes in our dataset, alongside the frequent use of seasonal vegetables, highlights a fundamental aspect of traditional Japanese cuisine—its intrinsic alignment with seasonality. The dataset reveals a pronounced preference for ingredients that are in season, reflecting a culinary culture that adapts to natural cycles and local availability. This seasonal orientation is not merely cultural but also serves critical nutritional and ecological functions.

Seasonal food consumption is a well-documented feature of traditional Japanese diets [47]. Seasonal vegetables provide enhanced freshness, superior nutrient density, and greater flavor, which contribute to dietary satisfaction and health outcomes [48]. In addition to nutritional gains, seasonal eating supports environmental sustainability by minimizing dependence on energy-intensive greenhouse production and long-distance transportation, thereby reducing the carbon footprint associated with food systems [49].

Our dataset’s explicit tagging corroborates these principles: a majority of vegetable ingredients correspond to well-known seasonal crops, including cabbages in spring, okras and cucumbers in summer, and root vegetables such as lotus roots in autumn. This adherence to seasonal cycles aligns with the traditional Japanese concept of *shun*, which emphasizes enjoying foods at their peak freshness and nutritional value [50].

The integration of seasonality also complements the health-promoting attributes of the Japanese diet. Seasonal vegetables typically provide higher vitamin bioavailability when harvested at their peak, due to optimal growing conditions and minimal post-harvest nutrient loss [51]. From a sustainability perspective, prioritizing seasonal vegetables reduces food waste and energy inputs associated with artificial growth conditions, aligning with broader goals of sustainable food systems [52]. This practice echoes findings from other studies documenting the environmental benefits of seasonal diets, which include reduced water use, land degradation, and greenhouse gas emissions [53].

### 4.4. Fermented Seasonings as Nutritional and Ecological Substitutes

The functional dominance of fermented seasonings in Japanese cuisine is quantitatively reflected in their displacement of discretionary additives. In our dataset, mirin (*n* = 22) substantially reduced added refined sugar: of the 39 recipes originally containing sugar, only 9 retained it after incorporating mirin—a 77% reduction. This substitution leverages mirin’s fermented sweetness and aligns with WHO sugar-reduction guidelines [54]. Similarly, soy sauce (*n* = 52) displaced added salt in 65% of salt-dependent recipes (72 → 25), offering equivalent umami depth with less salt per serving. This reduction is supported by research showing that soy peptides enhance salt taste perception, allowing reduced salt without loss of flavor [55]. Miso showed even greater salt-displacement efficacy, with only 1 of 7 miso-based recipes retaining added salt. Although the evidence for sugar reduction via soy-based seasonings is limited, the umami taste enhancement effect may contribute indirectly to reduced sugar usage by improving overall flavor complexity, which warrants further investigation.

Nutritionally, this displacement is complemented by fermentation-derived bioactives that offer additional health benefits. Fermentation enhances nutrient bioavailability, supports blood pressure regulation, promotes a more diverse gut microbiota, and produces compounds with antioxidant and anti-inflammatory properties [31,56,57]. These physiological effects occur alongside improved sensory qualities, as microbial fermentation generates a broad spectrum of volatile compounds that enrich the depth and complexity of flavor.

From an environmental perspective, fermentation efficiently converts underutilized grains and legumes into nutrient-dense flavorings through relatively low-energy, bio-friendly processes rather than energy-intensive methods [58]. Furthermore, by-products such as soybean residue (okara) are valorized—repurposed into fertilizer, animal feed, or higher-value bioproducts—significantly reducing waste [59].

### 4.5. Limitations

This study has four main limitations. First, nutritional composition (e.g., protein, vitamins, minerals) was not analyzed per dish, as recipes included ingredient lists without standardized nutritional profiles. Second, Japanese cuisine is characterized by strong regional diversity, yet national media may limit the research’s ability to reflect local dietary differences. Third, the demographic characteristics of the program’s audience and recipe users (e.g., age, socioeconomic status, and education) are unknown, which may limit the dataset’s representativeness of general Japanese home cooking practices. Finally, although Today’s Cooking offers detailed data on individual dishes, it remains unclear how these dishes are combined and consumed together in real-life meal settings. Therefore, future studies should conduct detailed nutritional profiling of dishes, incorporate region- and demographic-specific analyses, and employ real-time dietary recording methods to capture actual meal combinations.

## 5. Conclusions

This study offers a novel contribution to sustainable diet research by integrating diverse data sources beyond conventional academic materials. Specifically, we utilized recipe data from NHK’s nationally broadcast cooking program, Today’s Cooking, to explore potential insights into dietary practices as reflected in the recipes. This methodological innovation grounds the review in culturally embedded, widely disseminated recipes, offering insights that may inform connections between theoretical dietary models and household dietary practices. Such an approach offers a replicable framework for future studies seeking to align public-facing food media with academic inquiry into sustainable food systems.

Our findings affirm that contemporary home-cooked Japanese cuisine embodies a functional synthesis of nutrition, environmental stewardship, economic viability, and cultural continuity. This sustainability-oriented dietary paradigm is operationalized through several key practices: strategic protein sourcing that prioritizes low-trophic marine species and legume-based proteins; adherence to seasonal food procurement; flavor enhancement through fermentation rather than high resource inputs; and reliance on energy-efficient cooking methods. As a whole, these features reflect a culturally rooted approach to food that meets multiple dimensions of sustainability while preserving time-honored culinary traditions.

Nevertheless, the long-term viability of Japan’s sustainable dietary model faces growing challenges amid globalization and shifting consumption norms. National survey data (2003–2015) reveal a marked decline in traditional “plant and fish” dietary patterns, paralleled by increased intake of bread, dairy, oils, and ultra-processed foods [60]. In addition, two critical challenges warrant attention. First, Japan’s high per capita seafood consumption, while beneficial to public health due to its nutritional profile, places growing pressure on marine ecosystems. Overexploitation and habitat degradation necessitate more robust fisheries management and the development of alternative protein sources to ensure ecological sustainability. Second, high salt intake in the Japanese diet remains a persistent health concern, consistently linked to increased risks of hypertension and cardiovascular disease. Despite national dietary guidelines promoting salt reduction and specifying gender-specific daily intake targets, along with the inclusion of low-salt recipes on widely disseminated programs like NHK Today’s Cooking, sustained food reformulation and targeted public health interventions are still critically necessary. At the same time, since NHK Today’s Cooking recipes may not fully correspond to actual daily food practices, further research is needed to assess real-world dietary behavior.

To address these emerging pressures, longitudinal analyses of dietary practices and nutrient trends are important and warrant further investigation. Moreover, future research must move beyond compositional analysis to explore the practical, social, and cultural determinants of dietary behavior. This includes evaluating the extent to which traditional food practices can be sustained amid the demands of modern, convenience-oriented lifestyles. Identifying culturally resonant, feasible strategies that align with both sustainability goals and contemporary life will be critical to guiding long-term dietary transitions in Japan and beyond.

## Figures and Tables

**Table 1:** Ingredient-based classification of dishes from NHK Today’s Cooking (*n* = 120).

Ingredient Category	Number of Recipes (*n*)	Percentage (%)
Pork	40	33.3
Fish and Seafood	23	19.2
Chicken	21	17.5
Beef	3	2.5
Meatless	19	15.8
Non-Dish Items	18	15.0

Note: “Non-dish Items” include recipes for desserts and sauces that are not considered standalone main dishes. Four recipes contained both pork and fish as primary ingredients and were counted in each applicable category.

**Table 2:** Classification of legume-based preparations in NHK Today’s Cooking (*n* = 120).

Legume Type	Preparation	Protein Pairing	Count	Notes
Soy Products	Tofu	With beef	1	
		With chicken	1	
		With fish/seafood	1	1 dish contains miso
		Meatless	2	1 dish contains miso
	Tofu subtotal		5	
	Natto	With pork	1	
	Natto subtotal		1	
	Miso	With pork	2	
		With fish/seafood	2	1 dish contains tofu
		Meatless	2	1 dish contains tofu
		Non-dish item (seasoning)	1	
	Miso subtotal		5	Excluded 2 dishes with overlapping tofu
Other legumes	Non-soy legumes	Green beans	2	
		Broad beans	1	
		White kidney beans	1	
	Other legumes subtotal		4	
Total unique dishes			15	Excluded combo dishes

**Table 3:** Frequency of seasonings used in NHK Today’s Cooking dishes (*n* = 120).

Category	Seasoning	Frequency	% Dish Penetration	Biochemical Signature
Fermented	Soy sauce	52	43.3%	Glutamates, peptides
	Sake/Wine	47	39.2%	Ethanol
	Vinegar	23	19.2%	Acetic acid
	Mirin	22	18.3%	Ethanol, reducing sugars
	Miso	7	5.8%	Isoflavone glucosides
Non-Fermented	Salt	72	60.0%	Sodium chloride
	Pepper	56	46.7%	Piperine
	Sugar	39	32.5%	Sucrose

Note: One recipe may include more than one seasoning; therefore, total counts exceed the number of recipes analyzed.

**Table 4:** Pickled items identified in NHK Today’s Cooking recipes (*n* = 120) and their preservation characteristics.

Pickle Type	Preservation Agents	Shelf Life
Honey-pickled plum	Shochu, Coarse salt, Honey	1 year.
Ginger variants	Vinegar, Salt, Sugar	6 months.
Traditional pickled plum	Shochu, Coarse salt	1 year.
Taiwanese tea-infused plum	Rum, Coarse salt, Tea polyphenols	1 month.
Mixed summer vegetable pickles	Vinegar, Salt, Sugar	Not specified.

**Table 5:** Frequency of cooking methods in NHK Today’s Cooking recipes (*n* = 102).

Cooking Method	Frequency	Prevalence (%)
Simmering/Boiling	54	52.9%
Pan-frying	42	41.2%
Stir-frying	37	36.3%
steaming	18	17.6%
Deep-frying	10	9.8%
No-cooking or reheated	9	8.8%
baking	2	2.0%
pickling	2	2.0%
Total mentions	174	-

Note: 18 non-dish items (including dressings and desserts) were excluded from the initial 120 recipes (see Table 1).

## Data Availability

All data supporting the findings of this study are available within the article and its Supplementary Materials.

## Supplementary Materials

The following supporting information can be downloaded at: https://www.mdpi.com/article/10.3390/nu17162712/s1, Table S1: Recipe Database; Table S2: Sample.

## Abbreviations

The following abbreviations are used in this manuscript:

SHDs	Sustainable Healthy Diets
SDGs	Sustainable Development Goals
FAO	Food and Agriculture Organization
WHO	World Health Organization
UNESCO	United Nations Educational, Scientific and Cultural Organization
OECD	Organisation for Economic Co-operation and Development
MHLW	Ministry of Health, Labour and Welfare
MAFF	Ministry of Agriculture, Forestry and Fisheries
JPHC	Japan Public Health Center
